# Numerical Simulation of Crack Propagation in Concrete with Prefabricated Array Fractures Based on the Discrete Element Method

**DOI:** 10.3390/ma19153218

**Published:** 2026-07-28

**Authors:** Haiying Mao, Jun Zhen, Zuodong Zhou, Yaohui He, Xianzheng Zhu, Wenbing Zhang, Shuyang Yu

**Affiliations:** 1 Tianjin University of Technology, Tianjin 300384, China; 2 Guangxi University of Science and Technology, Liuzhou 545000, China; 3 School of Transportation and Civil Engineering, Nantong University, Nantong 226019, China; 4 College of Ocean Science and Engineering, Shanghai Maritime University, Shanghai 201306, China

**Keywords:** discrete element method, concrete, prefabricated fracture, uniaxial compression, crack propagation

## Abstract

Concrete readily develops cracks under service loads, which poses severe risks to the overall safety of engineering structures. In this work, the discrete element method (DEM) integrated with PFC2D 5.0 numerical software is adopted to construct a mesoscale concrete numerical model containing pre-existing internal fractures, and uniaxial compressive loading simulations are subsequently carried out. Unlike previous studies that predominantly examined isolated fracture parameters, this work systematically investigates the coupled effects of fracture inclination angle, length, and quantity on crack propagation mechanisms at the mesoscale, and for the first time establishes a quantitative relationship between microcrack spatial distribution patterns and macroscopic mechanical degradation. Parametric analyses are performed to quantify the influences of fracture geometric characteristics, including fracture inclination angle (30°, 45°, 60°), fracture length (short, long and extra-long), fracture quantity (4, 8 and 16), as well as the comparison between intact and fractured concrete specimens. The fracture quantities of 4, 8, and 16 are selected to represent low, medium, and high levels of initial defect density within the concrete matrix, corresponding to approximately 1%, 2%, and 4% of the total specimen area, respectively, thereby enabling a systematic investigation into the progressive deterioration of mechanical performance with increasing internal damage severity. The whole evolution process of crack initiation, crack propagation and ultimate failure patterns of concrete is systematically explored. Numerical results reveal that specimens with larger fracture angles exhibit higher compressive strength yet generate abundant newly formed microcracks, whereas low-angle prefabricated fractures are prone to triggering abrupt brittle failure. Specimens embedded with shorter fractures achieve superior mechanical strength and develop denser, more intensive microcrack distributions; in contrast, long pre-existing fractures drastically degrade compressive strength while limiting the generation of secondary cracks. Reducing the number of internal defects simultaneously improves compressive strength and expands the coverage range of the induced fracture network. Specimens with 16 prefabricated fractures deliver the weakest mechanical performance, owing to the excessively high initial defect density inside the matrix. In comparison with fractured samples, intact concrete without pre-set fractures achieves better comprehensive performance in terms of compressive strength, deformation compatibility and uniform microcrack development. A core conclusion drawn from this study is that the total quantity of microcracks cannot serve as a direct indicator to evaluate the damage degradation degree of concrete; instead, the spatial distribution pattern of microcracks dominates the deterioration level. Evenly scattered microcrack populations maintain relatively high residual strength, whereas the concentrated coalescence of microcracks into continuous penetrating macrocracks leads to an abrupt decline in structural load-carrying capacity. The findings of this research can provide theoretical references for stability evaluation and safety diagnosis of defective concrete structures in practical engineering.

## 1. Introduction

Concrete, characterized by its intrinsic multiphase composition comprising cement paste, aggregates, and interfacial transition zones, exhibits a remarkable combination of high compressive strength, favorable workability, and economic viability—attributes that collectively underpin its ubiquitous application across a diverse array of civil engineering infrastructures, ranging from high-rise buildings and long-span bridges to subterranean tunnels and hydraulic structures [1,2]. With global consumption approaching nearly 3 billion tons annually—a figure that more than doubles the combined production of steel, wood, plastics, and aluminium—concrete has undeniably cemented its status as the most extensively utilized manufactured material in modern construction practice worldwide [3,4]. Nevertheless, a substantial body of research has consistently demonstrated that concrete is inherently susceptible to cracking when subjected to uncontrolled internal stresses, abrupt temperature fluctuations, or prolonged cyclic loading—a susceptibility that represents a perennial challenge within the field of structural engineering, as the propagation of such defects can critically undermine both load-bearing integrity and long-term durability [5,6]. The deterioration process typically follows a progressive sequence, initiating with the coalescence of microstructural damage into discrete microcracks, which subsequently interconnect and propagate through the matrix, eventually giving rise to macroscopic fissures that often culminate in a brittle, catastrophic rupture mode [7]. Furthermore, concrete inherently contains defects or cracks. Local stress concentration occurs at the crack tip. This causes the material to enter a large-scale yield state. However, due to the limitations of small-scale yielding conditions, linear elastic fracture mechanics cannot be applied [8]. Therefore, traditional theoretical analysis alone is no longer sufficient for research needs. How to systematically and comprehensively analyse the fracture behaviour of concrete has become a research direction.

Despite extensive research on concrete fracture behaviour, existing studies have predominantly focused on single-factor analyses, such as isolated fracture inclination angles or individual defect sizes. The coupled effects of multiple geometric parameters (inclination angle, length, and quantity) on mesoscale crack propagation mechanisms remain insufficiently understood. Furthermore, the relationship between microcrack spatial distribution patterns and macroscopic mechanical degradation has not been quantitatively established. These knowledge gaps limit the accuracy of stability evaluation and safety diagnosis for defective concrete structures in practical engineering. To address these limitations, this study employs the discrete element method (DEM) integrated with PFC2D 5.0 numerical software to construct a mesoscale concrete model with pre-existing internal fractures. A systematic parametric analysis is conducted to investigate the coupled influences of fracture geometric characteristics on crack initiation, propagation, and coalescence under uniaxial compression. The novelty of this work lies in: (1) simultaneously considering three key fracture geometric parameters (inclination angle, length, and quantity) within a unified numerical framework; (2) revealing the competitive relationship between microcrack quantity and spatial distribution in governing macroscopic mechanical performance; and (3) establishing a quantitative criterion linking microcrack distribution patterns to structural degradation levels.

Experimental methodologies, which constitute the foundational pillar of empirical materials research, are conventionally prioritized in the initial investigative stages, as they offer the distinct advantage of physically replicating the mechanical responses of loaded specimens under compressive stresses, thereby yielding empirical data of high authenticity and practical relevance for studying crack development. For example, Ricardo Antonio Barbosa et al. [9] used uniaxial compression tests to study the influence of crack orientation and crack number on the compressive strength of concrete. Gou et al. [10] used uniaxial compression tests to study the relationship between concrete crack development and the number of freeze–thaw cycles. Guo et al. [11] used uniaxial compression tests to study the effects of interface inclination angle, defect inclination angle, and strength ratio on crack development in double-defect rock-concrete specimens. Liu et al. [12] used uniaxial compression tests to study the influence of grinding-type steel fibres on the maximum crack width of concrete under compression. Liu et al. [13] used uniaxial compression tests to study the effects of specimen shape and carbon fibre use on the number of cracks developed in concrete. Li et al. [14] used uniaxial compression tests to study crack development patterns in seawater sea-sand ultra-high performance concrete and freshwater river-sand ultra-high performance concrete after five years of sulfate attack.

Experimental studies can directly observe crack development on concrete surfaces. The results have high authenticity and reliability. They are consistent with actual engineering stress conditions. In addition, experiments can account for real-world factors such as material mix proportions, interface characteristics, and environmental conditions. The conclusions obtained can be directly used for structural design and safety evaluation.

However, experimental studies also have limitations. Small internal cracks in concrete are difficult to observe. Testing equipment is costly and requires long periods of time. This is especially evident when simulating complex conditions. Moreover, once a specimen is damaged during testing, it is difficult to repeatedly verify the same state.

Theoretical analysis is another important research approach. It can explain experimental phenomena. It can also establish mechanical models. It can predict crack evolution and failure patterns in concrete under uniaxial compression. Therefore, researchers have carried out corresponding model construction and formula derivation. These efforts are based on theories such as continuum mechanics, damage mechanics, and fracture mechanics. For example, Peter Grassl et al. [15] studied the mechanical behaviour of concrete under uniaxial compression. They used a hardening law based on volumetric plastic strain and plasticity theory. Mazzotti C et al. [16] proposed a nonlinear creep-damage coupled model for concrete under uniaxial compression. They used a strain decomposition assumption to separate creep and damage contributions. Kurumatani et al. [17] studied an isotropic damage model for quasi-brittle materials. They used fracture mechanics theory, a cohesive crack model, and a modified von Mises equivalent strain criterion. Their work enabled simulation of crack propagation in concrete. Luís A. G et al. [18] studied crack evolution in steel fibre-reinforced concrete during loading. They used isotropic damage theory, an implicit-explicit integration algorithm, and finite element coupling theory. Li et al. [19] studied quasi-brittle crack propagation mechanisms in concrete. They used the discrete lattice spring model (DLSM), mesomechanical constitutive theory, and material heterogeneity and geometric non-uniformity characteristics. Sun [20] studied the softening behaviour and crack evolution mechanisms of concrete at high temperatures. He used dimensional analysis theory and continuum damage mechanics theory.

Theoretical analysis can quantitatively derive many useful key indicators through formulas. It is a method that can explain crack initiation and propagation from a mechanical perspective. It is not constrained by specimen preparation or testing equipment. Variables can be adjusted conveniently. It can quickly clarify the influence of a single factor. It has low computational cost and high research efficiency. It can also provide a theoretical reference framework for experiments and numerical simulations.

Despite their analytical rigor, most prevailing theoretical frameworks are predicated upon drastically simplified solutions that treat concrete as an idealized homogeneous continuum, thereby conveniently neglecting the inherent heterogeneous, multiphase nature of the material—specifically, the distinct mechanical contrasts among aggregates, mortar, and the weak interfacial transition zones—a simplification that fundamentally precludes the faithful representation of critical mesoscale phenomena, such as the crack-arresting effect of coarse aggregates or the preferential propagation along weak interfaces. For complex conditions such as multiple interacting cracks and branched propagation, theoretical equations become very difficult to solve. It is hard to accurately describe the evolution of random cracks. Therefore, theoretical results inevitably deviate from the actual fracture characteristics of concrete.

Numerical simulation is an effective research tool. It can reproduce the entire loading process of concrete. It can track mesoscale crack propagation paths. It can reveal damage and failure mechanisms under uniaxial compression. It is an indispensable core component in the study of concrete mechanical properties. Compared with laboratory tests, numerical simulation can avoid experimental limitations. It can accurately capture the whole process of crack initiation, propagation, and coalescence. This process is difficult to observe directly during compression. It can efficiently complete mechanical response analysis and model validation.

Therefore, researchers have carried out extensive simulation analyses and model optimisation studies. These efforts focus on the deformation characteristics, crack evolution behaviour, and overall failure mechanisms of concrete under uniaxial compression. Both domestic and international researchers have contributed. They rely on two mainstream numerical methods: the finite element method (FEM) and the discrete element method (DEM).

The finite element method can accurately characterise the macroscopic mechanical response and overall softening damage characteristics of concrete as a continuous medium. It is suitable for simulating macroscopic deformation and overall failure processes of concrete under uniaxial compression. For example, Bhowmick [21] studied brittle fracture and crack propagation in solids. He used the cell-based smoothed finite element method (CS-FEM) combined with a phase-field model. His work covered complex behaviours such as crack initiation, coalescence, and branching. Toyama et al. [22] studied fatigue crack propagation in concrete mortar under cyclic contact loading. They used the finite element method. Zhang et al. [23] studied mixed-mode I-II unstable crack propagation in concrete. They used a finite element method with continuous nodal stresses. Sun et al. [24] studied three-dimensional fracture processes in brittle-like materials. They used continuum damage mechanics and the finite element method. Rombach et al. [25] studied crack propagation behaviour in reinforced concrete beams without stirrups. They used the extended finite element method (XFEM) under bending and shear loading. Nguyen et al. [26] studied localisation failure in quasi-brittle materials. They used low-order finite element elements.

The discrete element method (DEM) can effectively characterise the separation, sliding, and cracking behaviour between mesoscale particles in concrete. It can accurately reproduce the internal crack evolution process of concrete under compression. It provides reliable numerical support for studying the mechanical properties and failure mechanisms of concrete under uniaxial compression.

For example, Zhu et al. [27] used the discrete element method to study a three-dimensional hydraulic fracturing model. Their model considered the coupling of continuous fields, crack opening, pore seepage, and fracture seepage fields. Du et al. [28] used the discrete element method to study the damage process of roller-compacted concrete under freeze–thaw cycles and triaxial compression. Hamelin et al. [29] used the discrete element method to study the effect of density on the elastic and fracture behaviour of particles. Zhang et al. [30] used the discrete element method to study the propagation behaviour of built-in inclined circular three-dimensional cracks under uniaxial compression. Zhu et al. [31] used the discrete element method to simulate mesoscale crack propagation in concrete under tensile stress. They studied the mesoscale damage evolution of concrete. J. D. Riera et al. [32] used the discrete element method to evaluate its capability in predicting the static three-dimensional compression behaviour of concrete. M. Krzaczek et al. [33] used the discrete element method to study the mechanical behaviour and crack propagation mechanisms of partially saturated concrete under quasi-static uniaxial compression.

From the above studies, it can be seen that the discrete element method has significant advantages in studying concrete crack propagation. It can simulate crack propagation under various loading conditions. These include uniaxial compression and triaxial compression. It can provide prediction results for subsequent experimental tests. In addition, the discrete element method can consider concrete under different environmental conditions. These include freeze–thaw cycles and high temperatures. It can study concrete crack propagation from various aspects.

Therefore, considering these advantages, this study finally adopts the discrete element method to investigate crack propagation in concrete under uniaxial compression.

Despite the extensive body of research reviewed above, several critical gaps remain in the current understanding of concrete fracture behaviour under uniaxial compression.

From the experimental perspective, existing studies such as those by Barbosa et al. [9], Gou et al. [10], and Guo et al. [11] have successfully documented crack development patterns and established qualitative relationships between fracture geometry and compressive strength. However, these experimental investigations are inherently limited by their inability to capture the internal three-dimensional crack evolution process; small internal microcracks remain unobservable, and the destructive nature of testing precludes repeated verification of identical specimen states under varying conditions.

From the theoretical perspective, models proposed by Grassl et al. [15], Mazzotti and Savoia [16], and Kurumatani et al. [17] have provided valuable analytical frameworks for predicting macroscopic mechanical responses. Nevertheless, these continuum-based approaches typically idealise concrete as a homogeneous material, thereby neglecting the heterogeneous multiphase characteristics of aggregate, mortar, and the interfacial transition zone. Consequently, they fail to capture mesoscale phenomena such as aggregate crack resistance and interface-preferred cracking, and become mathematically intractable when addressing multiple interacting cracks or branched propagation paths.

From the numerical perspective, while FEM-based studies by Bhowmick [21], Toyama et al. [22], and Rombach et al. [25] have advanced the simulation of macroscopic deformation and overall failure processes, they encounter significant challenges in reproducing the discrete, discontinuous nature of crack initiation and coalescence at the mesoscale.

Although DEM simulations by Zhu et al. [27], Du et al. [28], and Zhang et al. [30] have demonstrated superior capability in tracking internal crack evolution and particle-scale damage accumulation, existing DEM studies have predominantly focused on isolated fracture parameters—examining either inclination angles or defect sizes in isolation—without systematically investigating the coupled effects of multiple geometric parameters. Furthermore, the quantitative relationship between microcrack spatial distribution patterns and macroscopic mechanical degradation has not been established in the existing DEM literature.

To address these limitations, the present study employs the discrete element method (DEM) integrated with PFC2D numerical software to construct a mesoscale concrete model containing pre-existing internal fractures. Unlike previous investigations that examined single-factor effects in isolation, this work simultaneously considers three key fracture geometric parameters—inclination angle, length, and quantity—within a unified numerical framework.

The novelty of this study lies in: (1) conducting a systematic parametric analysis to reveal the coupled influences of fracture geometric characteristics on crack initiation, propagation, and coalescence under uniaxial compression; (2) establishing, for the first time, a quantitative criterion linking microcrack spatial distribution patterns to structural degradation levels; and (3) demonstrating that the spatial distribution pattern of microcracks, rather than their total quantity, dominates the macroscopic mechanical deterioration.

These contributions provide a more comprehensive theoretical basis for stability evaluation and safety diagnosis of defective concrete structures in practical engineering.

Recent years have seen active research on crack development in concrete under uniaxial compression. Yu et al. [34] studied how loading speed and sample size affect the failure of basalt fiber-reinforced lightweight concrete. Ren et al. [35] used a new damage model to simulate concrete fracture and examined how the interfacial transition zone affects crack patterns. Ibrahim Albaijan et al. [36] applied eleven machine learning algorithms to predict nano-silica concrete compressive strength. Wen et al. [37] built 3D concrete models from CT scan images and simulated uniaxial compression with prediction errors below 15%. Xu et al. [38] investigated recycled aggregate concrete by pre-coating brick-concrete aggregate with composite cement materials. These studies cover experiments, computer models, machine learning, and sustainable materials.

## 2. Principles of PFC

### Principle of Crack Simulation in PFC

**Figure 1:**
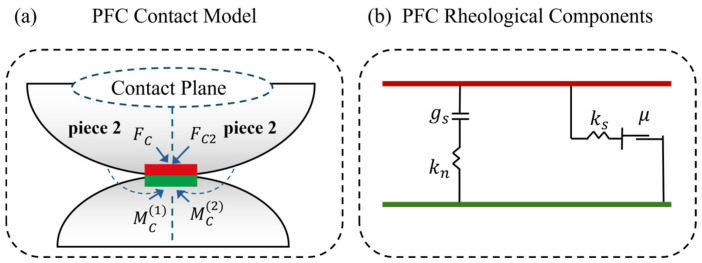
Particle contacts in PFC. (**a**) Contact Plane (**b**) Rheological Components.

With the discrete element method, concrete is regarded as a discontinuous medium composed of rigid particles. The contact and bonding between particles represent the mesoscale structure of the material. The model consists of three components: particles, contacts, and walls. The particles represent the mesoscale structures such as aggregate and mortar. The walls serve as loading platens or boundaries to apply loads. The contacts are responsible for transmitting forces and moments. The parallel bond model is commonly used. This model can transmit both forces and moments. It also enables concrete to sustain bending moments. This is consistent with the cementitious characteristics of concrete. The schematic diagram of the principle is shown in Figure 1.

The force calculation is performed through an explicit iterative cycle. At each step, the contacts between particles are first detected. Then, according to the contact constitutive law, the normal and shear contact forces are calculated from the relative displacements. Next, the acceleration, velocity, position, and rotation of each particle are updated using Newton’s second law. These updates are based on the resultant force and resultant moment. After that, all contact states are refreshed. The code checks whether any bonds have broken. It also checks whether new cracks have been generated. This process is repeated continuously. It continues until the specified displacement or loading condition is reached. This approach does not require pre-defined cracks. It can naturally capture stress redistribution. It also captures crack initiation, propagation, and coalescence.

In the simulation process, the mesoscale model of aggregate and mortar is first constructed. This is done by randomly placing particles with different sizes. The mesoscopic parameters are then calibrated. This calibration is based on the macroscopic mechanical properties of concrete. The actual concrete parameters are input into the model. This ensures that the relevant parameters of the simulated concrete are consistent with the test data.

After the model is generated, the loading operation begins. During loading, a constant velocity or constant stress is generally set first. The load is then applied through the walls. In this process, the external force first acts on the boundary particles. It is then transmitted to the interior. A stress field is formed inside the specimen. During the loading process, PFC continuously simulates the propagation of concrete cracks. At the same time, PFC monitors the number, type, and distribution changes in cracks in real time.

## 3. Numerical Model and Parameters

### 3.1. Setting of Mesoscopic Parameters

This study employs the Linear Parallel Bond Model (LPBM) to simulate the mesoscale mechanical behavior of concrete. Considering that concrete is a multiphase composite material composed of coarse aggregates and a cement mortar matrix, the model distinguishes between aggregate particles and mortar particles, assigning different mesoscale mechanical parameters to each phase to reflect the heterogeneity of the actual material. All parameters in the model adopt the International System of Units (SI), with length in meters (m), force in Newtons (N), and stress and modulus in Pascals (Pa).

The particle effective elastic modulus, Emod, serves as the effective elastic modulus in the PFC deformability formulation, determining the contact stiffness together with the particle radius. In this study, the Emod for mortar particles is set to 1.30 × 10^10^ Pa (approximately 13 GPa), while that for aggregate particles is set to 5.55 × 10^10^ Pa (approximately 55.5 GPa). The stiffness of the aggregate phase is approximately 4.3 times that of the mortar phase, which is consistent with the mechanical properties of actual concrete where aggregates (such as granite or limestone, with elastic moduli typically ranging from 50 to 80 GPa) are significantly stiffer than the cement mortar matrix (with elastic moduli typically ranging from 10 to 30 GPa).

The parallel bond elastic modulus, Pb_emod, is kept consistent with the contact modulus of the corresponding particle phase, i.e., Pb_emod is set to 1.30 × 10^10^ Pa for the mortar phase and 5.55 × 10^10^ Pa for the aggregate phase. This configuration ensures stiffness continuity before and after bonding, preventing numerical oscillations caused by a mismatch between contact stiffness and bond stiffness.

The normal-to-shear stiffness ratio, Kratio, defined as the ratio of normal stiffness to shear stiffness, has a significant influence on the Poisson’s ratio of the material. The mortar phase is assigned a Kratio of 2.0, while the aggregate phase is assigned 1.5. The lower stiffness ratio of the aggregate phase reflects its relatively stronger shear stiffness characteristics. The linear contact and parallel bond employ the same stiffness ratio to ensure consistency in the mechanical response.

The parallel bond tensile strength, Pb_ten, governs the tensile failure threshold of the bonded interface. The mortar phase is assigned a Pb_ten of 5.0 × 10^6^ Pa (5 MPa), while the aggregate phase is assigned 2.0 × 10^7^ Pa (20 MPa). The tensile strength of the aggregate phase is approximately four times that of the mortar phase, reflecting the physical reality that the bonding strength between aggregate particles is significantly higher than that of the mortar matrix.

The parallel bond cohesion, Pb_coh, controls the shear failure threshold of the bonded interface. The mortar phase is assigned a Pb_coh of 6.0 × 10^6^ Pa (6 MPa), while the aggregate phase is assigned 2.5 × 10^7^ Pa (25 MPa), maintaining a coordinated proportion with the tensile strength. The lower cohesion of the mortar phase causes cracks to tend to initiate and propagate within the mortar matrix and interfacial regions, which is consistent with the weak interfacial transition zone (ITZ) effect observed in mesoscale experiments of concrete.

The parallel bond friction angle, Pb_PFA, controls the inclination of the shear strength envelope. The mortar phase is assigned a friction angle of 45°, while the aggregate phase is assigned 40°. Both values fall within the typical friction angle ranges for rock and concrete materials. The slightly higher friction angle of the mortar phase compared to the aggregate phase reflects the interlocking effect of cement hydration products under shear action.

The damping coefficient, damp, is used in the local damping scheme of PFC to dissipate kinetic energy and facilitate static equilibrium. Both the mortar and aggregate phases are assigned a uniform damp value of 0.7. This value ensures stable convergence of the computation while effectively suppressing kinetic energy fluctuations in the particle system during quasi-static loading, yielding relatively smooth stress–strain curves.

The above parameter values are determined by referring to the physical and mechanical properties of some common concrete materials and are preliminarily estimated using empirical formulas. For example, approximate proportional relationships exist between the parallel bond tensile strength and the macroscopic tensile strength, as well as between the cohesion and the macroscopic compressive strength. Based on these relationships, the mesoscale parameters are preliminarily estimated. Since this study is a pure numerical simulation without corresponding physical experiments, the parameters have not been calibrated against experimental data, and the obtained results mainly reflect qualitative trends.

Although no measured data were available for calibration, the adopted parameter values are all within the reasonable ranges reported in the literature. They can effectively reflect the basic deformation and fracture characteristics of concrete. Since this study focuses on the relative comparison between different test conditions, all cases adopt the exact same parameter settings. Therefore, the absolute accuracy of the parameter values does not affect the validity of the comparative conclusions. The model can still approach the actual structural response of concrete to the greatest extent possible. The relevant parameters are presented in Table 1.

The mesoscopic parameters used here are physically rational and reliable. All indices are derived from widely accepted PFC simulation intervals for concrete, with the modulus and strength ratios of aggregate and mortar consistent with real material properties. This parameter set has been optimized via pre-simulation sensitivity tests to avoid numerical instability, and uniform parameters across all groups ensure unbiased comparative results. The relevant parameters are presented in Table 1.

### 3.2. Computational Model and Specimen Dimensions

**Figure 2:**
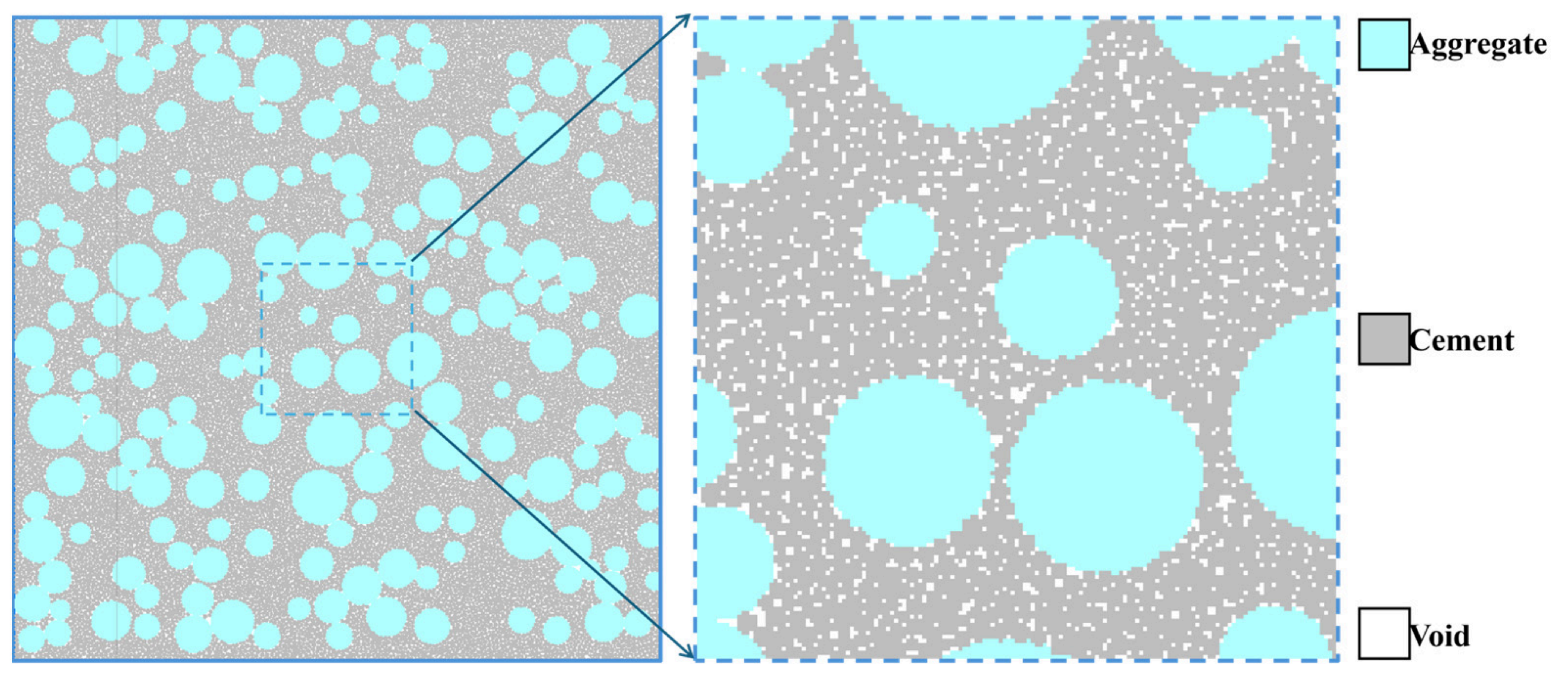
Mesoscale model of concrete.

The model adopts a square specimen. The width and height are both 0.06 m. The specific geometry is shown in Figure 2. In the particle flow code (PFC), eight groups of spheres with different particle sizes are generated. The first seven groups correspond to coarse aggregate particles with different size ranges. These are used to simulate crushed stones or pebbles of varying sizes in actual concrete. The eighth group represents cement mortar particles. These serve as the matrix material filling the spaces between aggregates. Each group of particles is randomly distributed within the model area according to a certain gradation. Together, they form the mesostructure of concrete.

## 4. Results of Numerical Tests

### 4.1. Strength Characteristics of Concrete in the Tests

#### 4.1.1. Stress–Strain Curves

Based on multiple sets of stress–strain curves derived from the PFC numerical simulations, this study systematically investigates the effects of four distinct categories of test conditions on the mechanical performance of concrete under uniaxial compression, specifically examining: (1) fracture inclination angles (30°, 45°, and 60°), (2) fracture lengths (short, long, and very long), (3) fracture quantities (4, 8, and 16), and (4) a comparative analysis between specimens incorporating prefabricated fractures and those devoid of such initial defects.

A comprehensive comparison reveals that prefabricated fractures serve as initial defects. They alter the internal stress distribution, damage evolution, and load transfer paths at the mesoscale level. This ultimately leads to differences in macroscopic load-bearing performance. An overarching synthesis of the acquired numerical data reveals a consistent and systematic trend: increased fracture inclination angles, diminished fracture lengths, and reduced defect quantities are all unequivocally associated with elevated stress levels at equivalent strain magnitudes, whereas the structurally intact, defect-free specimens consistently exhibit the paramount load-bearing performance among all tested configurations. Detailed analyses for each case are presented below.

(1) Fracture inclination angle (30°, 45°, 60°)

**Figure 3:**
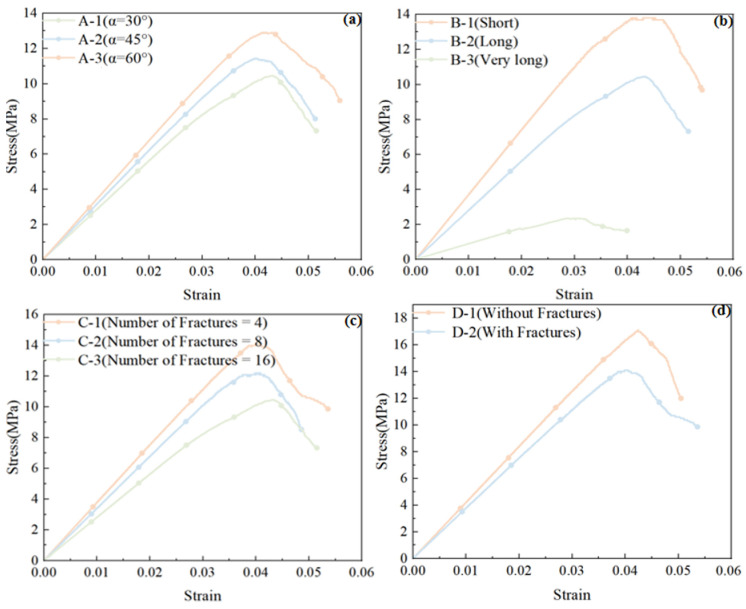
Stress–strain curves for specimens under different conditions: (**a**) fracture inclination angle; (**b**) fracture length; (**c**) number of fractures; (**d**) presence or absence of prefabricated fractures.

From the Group (a) curves in Figure 3, it can be observed that a larger fracture inclination angle results in a higher stress at the same strain level. Under uniaxial compression, the inclination angle directly determines the stress field distribution at the crack tip. When the inclination angle is 30°, the fracture plane is nearly horizontal. Under external loading, the fracture plane mainly experiences significant shear stress. At the same time, notable tensile stress concentration occurs at the crack tips. This easily induces shear slip along the fracture direction and wing crack propagation. As a result, the specimen undergoes local failure at a relatively low stress level.

As the inclination angle increases to 45°, the stress state changes. The ratio of shear stress to normal stress approaches a balance. The degree of tensile stress concentration is alleviated to some extent. The load level required for microcrack initiation increases accordingly. When the inclination angle reaches 60°, the fracture is closer to vertical. At this point, the external force mainly causes the fracture plane to close under compression. The tensile effect at the tips is significantly weakened. The stress distribution becomes more similar to that of an intact material. Therefore, the specimen can withstand a higher stress without instability failure.

From the perspective of mesoscale damage evolution, a small inclination angle accelerates damage propagation from the tips along the loading direction. This forms a through-going crack. A large inclination angle effectively suppresses this propagation tendency. The damage evolution becomes slower and more dispersed. The stress–strain curves from the simulations clearly demonstrate this trend. The peak stress of the 60° specimen is approximately 15% to 20% higher than that of the 30° specimen. This fully verifies that increasing the inclination angle improves the compressive performance of concrete.

(2) Fracture length (short, long, and very long)

From the Group (b) curves in Figure 3, it can be observed that a shorter fracture length results in a higher stress at the same strain level. The fracture length directly determines the degree of weakening of the effective load-bearing cross-section. It also determines the size of the stress concentration zone. For short fractures, the cutting effect on the matrix is limited. The stress concentration near the crack tip is confined to a very small zone around the tip. A large amount of intact matrix material surrounds this zone. This intact material effectively constrains the initiation and propagation of microcracks. During loading, the main damage still appears as uniform microcracking within the matrix. The overall stiffness of the specimen does not decrease significantly.

When the fracture length increases to “long”, the effective bearing area is noticeably reduced. The high-stress zones on both sides of the fracture and around the tips become closer to each other. Microcracks are more likely to propagate rapidly from the tips along the loading direction. They also connect with surrounding defects more easily. For “very long” fractures, the specimen is almost divided into two relatively independent load-bearing parts. The load transfer path changes fundamentally. The external force can only be transmitted through the narrow zones at the fracture ends. This causes extremely high stress concentration in these zones. Once microcracks initiate, they quickly coalesce into a through-going crack. The specimen exhibits obvious brittle failure characteristics.

The PFC simulation results provide quantitative evidence. Under the same strain condition, the peak stress of the long-fracture specimen is approximately 25% to 30% lower than that of the short-fracture specimen. For the very-long-fracture specimen, the peak stress reduction even exceeds 40%. This trend fully demonstrates that short fractures pose a much smaller threat to structural safety than long fractures.

(3) Number of fractures (4, 8, and 16)

From the Group (c) curves in Figure 3, it can be observed that a smaller number of fractures results in a higher stress at the same strain level. The number of fractures is a core parameter. It determines the density of internal defects in concrete. It also determines the degree of damage coupling effects. When only 4 fractures are distributed in the specimen, these defects are kept at a certain distance from each other. The stress concentration zones around each fracture tip are relatively independent. They are less likely to interfere with one another. At this stage, the matrix material can still form a relatively continuous load-transfer network. The external force is transmitted uniformly through the undisturbed regions. During loading, microcracks first initiate at individual fracture tips. However, their propagation is hindered by the surrounding intact matrix. The overall damage evolution is relatively slow. The specimen can undergo a long yield stage before failure occurs.

When the number of fractures increases to 8, the spacing between fractures decreases. The stress fields at adjacent fracture tips begin to overlap. The local high-stress zones connect into a continuous area. Multiple crack sources are activated simultaneously and quickly coalesce. This leads to a rapid reduction in the effective load-bearing area. When the number of fractures reaches 16, the specimen interior is almost divided into many small independent blocks by the fracture network. The original continuous load-bearing system completely collapses. The external force cannot be transmitted through an intact path. It can only rely on the narrow “bridging” zones between fractures. Extremely strong stress concentration occurs in these bridging zones. Microcracks penetrate instantaneously. The specimen enters the failure stage almost without a noticeable elastic phase.

The simulation data provide quantitative evidence. The peak stress of the 16-fracture specimen is only 60% to 70% of that of the 4-fracture specimen. Its stress–strain curve also shows a sharp drop after the peak. This indicates a more pronounced brittle behaviour. It can be concluded that an increasing number of fractures aggravates the degradation of concrete.

(4) Presence or absence of prefabricated fractures (with fractures vs. without fractures)

From the Group (d) curves in Figure 3, it can be observed that the stress of the fracture-free specimen is always higher than that of the fractured specimen at the same strain level. This comparison directly reveals the essential influence of initial defects on the compressive performance of concrete. For the intact specimen without fractures, the internal particles and parallel bonds remain continuous and complete during the initial loading stage (elastic stage). The external force is uniformly transmitted across the entire cross-section. There is no stress concentration or weak interface. At this stage, the stress–strain curve rises linearly. The elastic modulus is the highest. The overall stiffness and deformation resistance reach their optimum.

As the load increases, uniform microcrack damage gradually develops within the material. However, there is no pre-existing macroscopic fracture to guide the failure path. Therefore, the peak stress is the highest. The specimen can still maintain a certain degree of ductility after the peak. In contrast, the specimen with prefabricated fractures contains structural discontinuities from the very beginning of loading. Significant stress concentration occurs at the fracture edges even at very low stress levels. Even when the external force is far below the material strength, the local region near the fracture tip has already entered a non-linear state. This stress concentration disrupts the original uniform load-transfer system. As a result, the stress that the specimen can sustain at the same deformation is significantly reduced.

Taking the simulation results as an example, at a strain of 0.04%, the stress of the fracture-free specimen is approximately 1.0 to 1.2 times that of the fractured specimen. In addition, the elastic stage of the fractured specimen is significantly shortened. Microcracks at the fracture tips initiate at low stress levels. They then propagate rapidly along the loading direction. Overall, this results in a lower peak stress and a more brittle failure mode. Therefore, the superior performance of the fracture-free specimen originates from its complete structural continuity. Any form of prefabricated fracture fundamentally weakens the load-bearing capacity of concrete. This is the core reason why the mechanical performance of fractured specimens is always inferior to that of intact specimens.

(5) Comprehensive analysis and discussion

By synthesising the four types of test conditions, a general conclusion can be drawn. The geometric parameters and distribution characteristics of prefabricated fractures essentially regulate the development of mesoscale damage. They do so by altering the scale of internal defects, the degree of stress concentration, and the structural continuity of concrete. A larger number of defects leads to more severe degradation. A longer fracture length leads to more severe degradation. A smaller inclination angle also leads to more severe degradation. In all these cases, the internal load-bearing system of concrete is more seriously disrupted. Damage evolution accelerates accordingly. The macroscopic mechanical performance becomes progressively worse.

#### 4.1.2. Crack Number Evolution Curves

During the uniaxial compression loading process, the number of new microcracks inside the specimen continuously evolves with loading time. The rate of change and the final cumulative value directly reflect the intensity of damage development and the failure mode. Based on multiple sets of crack number–time curves obtained from PFC numerical simulations, this study examines the influence of initial fracture characteristics on the damage evolution process of concrete from four aspects. These are fracture inclination angle (30°, 45°, and 60°), fracture length (short, long, and very long), number of fractures (4, 8, and 16), and the presence or absence of prefabricated fractures.

The crack number–time curves under different test conditions show obvious differences. These differences are reflected in the initiation time of cracks. They are also reflected in the rate of crack growth. They are further reflected in the final cumulative number of cracks. It is worth noting that the final crack number does not have a simple corresponding relationship with the macroscopic load-bearing capacity of the specimen. Together, they reveal the dual regulation effect of initial defects on the mechanical response and failure process of concrete. Detailed analyses for each case are presented below.

(1) Fracture inclination angle (30°, 45°, 60°)

**Figure 4:**
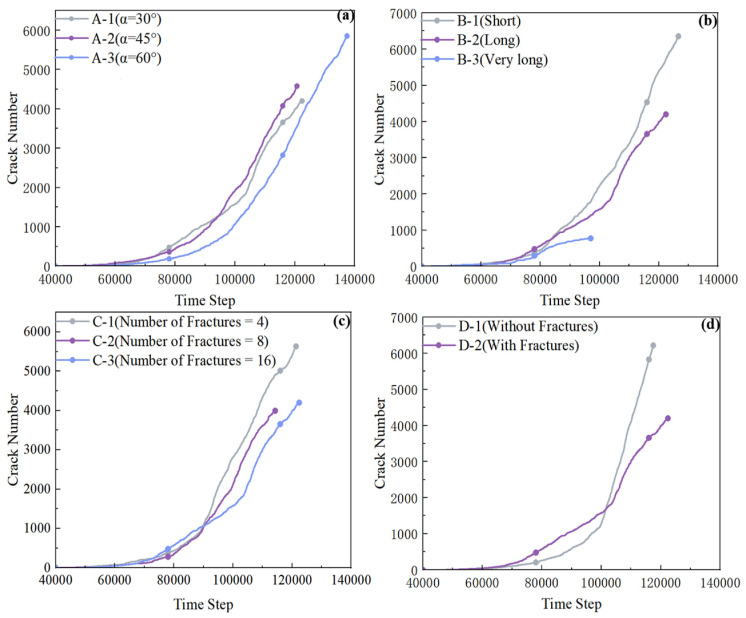
Crack number evolution curves for specimens under different conditions: (**a**) fracture inclination angle; (**b**) fracture length; (**c**) number of fractures; (**d**) presence or absence of prefabricated fractures.

From the final crack counts in Group (a) of Figure 4, it can be observed that the 60° specimen has the highest number of cracks. The 45° specimen ranks second. The 30° specimen has the lowest number of cracks. In terms of the crack growth rate, differences are also observed at different loading stages. At the initial stage of loading, the 30° specimen shows the fastest crack initiation rate. The 60° specimen shows the slowest rate. In the middle stage of loading, the rate of the 60° specimen increases rapidly. It surpasses the other two groups. It becomes the fastest among the three. At the same time, the rates of the 30° and 45° specimens tend to approach each other.

This pattern differs from the ranking of mechanical properties. The 30° specimen has the lowest final crack count. However, it has the fastest initiation rate at the initial stage. The reason is as follows. For a small inclination angle, significant tensile stress concentration occurs at the crack tip even at a low stress level. This promotes early initiation of microcracks. However, these early cracks quickly coalesce along the fracture direction. The specimen enters the failure stage within a relatively short period. The loading duration is short. There is insufficient time and stress conditions for more new cracks to initiate later. Therefore, the final total number is the lowest.

The 60° specimen shows the opposite trend. A large inclination angle weakens the tensile effect at the crack tips. At the initial stage, microcracks are difficult to initiate. The rate is the slowest. As the strain increases and the stress redistributes, damage begins to spread in multiple directions. At the same time, the specimen can sustain a higher load. It also experiences a longer deformation process. Therefore, the crack count accumulates rapidly in the middle stage. The final total number becomes the highest. The 45° specimen shows intermediate behaviour in all aspects.

The above results indicate that the number of cracks is not directly equivalent to the degree of degradation. The 60° specimen produces the most microcracks. However, these cracks are relatively dispersed. They do not form a through-going failure. The 30° specimen produces fewer cracks. Yet these cracks are highly concentrated at the fracture tips. They quickly coalesce. This leads to a more brittle macroscopic failure.

(2) Fracture length (short, long, and very long)

From the final crack counts in Group (b) of Figure 4, it can be observed that the short-fracture specimen has the highest number of cracks. The long-fracture specimen ranks second. The very-long-fracture specimen has the lowest number of cracks. In terms of the crack growth rate, the short-fracture specimen shows the fastest rate. The long-fracture specimen shows an intermediate rate. The very-long-fracture specimen shows the slowest rate. Moreover, the rate of the very-long-fracture specimen tends to approach zero at the later stage of loading.

Short fractures cause the least weakening of the macroscopic load-bearing capacity. However, they trigger the largest number of new microcracks during the loading process. The reason is as follows. The stress concentration zone at the tip of a short fracture is limited in size. The initiated microcracks are continuously hindered by the surrounding intact matrix during propagation. They cannot easily coalesce into a through-going crack. The loading energy is continuously converted into dispersed new cracks. Therefore, the crack count accumulates continuously. The growth rate remains at a relatively high level throughout.

For the long-fracture specimen, the initial fracture itself has already significantly weakened the cross-section. The high-stress zone at the tips is larger. Once microcracks initiate, they easily propagate rapidly along the tip direction. They also connect with the main fracture quickly. Therefore, the total number of new cracks is relatively small. The growth rate is relatively moderate.

For the very-long-fracture specimen, the initial fracture almost runs through the entire specimen. The structural continuity inside the specimen has been severely damaged. During loading, the external force is mainly transmitted through the extremely narrow zones at the fracture ends. These zones undergo local failure even at a relatively low strain level. However, the subsequent damage evolution is dominated by further opening and sliding of the existing fracture. It is difficult to initiate a large number of new independent microcracks in the intact matrix. Therefore, the crack count increases slightly at the initial stage of loading. It then tends to stagnate. The growth rate eventually approaches zero. This indicates that the damage evolution has shifted from initiating new cracks to propagating and coalescing the existing cracks.

In summary, a shorter fracture length leads to a greater number of new microcracks and a faster initiation rate. However, the damage caused by these cracks is relatively limited. In contrast, a longer fracture length results in fewer new cracks and a slower initiation rate. However, the existing fracture itself has already severely compromised the structural continuity. This is sufficient to significantly reduce the load-bearing capacity.

(3) Number of fractures (4, 8, and 16)

From the final crack counts in Group (c) of Figure 4, it can be observed that the specimen with 4 fractures has the highest number of cracks. The specimen with 8 fractures ranks second. The specimen with 16 fractures has the lowest number of cracks. In terms of the crack growth rate, the three groups show roughly comparable rates throughout the entire loading process. No obvious stage-dependent differences are observed among them.

When the initial number of fractures is 4, there are relatively large intact regions within the specimen. The matrix is relatively continuous. Under external loading, there is sufficient space for new microcracks to initiate and propagate. The stress fields generated around each initial fracture tip are independent of one another. Microcracks gradually develop within each affected zone. The total number increases linearly with strain. The final cumulative count is the highest among the three groups.

When the initial number of fractures is 8, the spacing between fractures decreases. Stress field superposition occurs in some regions. Microcracks initiate. However, they also coalesce with adjacent fractures more quickly. During the coalescence process, some new cracks merge into larger cracks. These merged cracks are no longer counted individually as microcracks. Therefore, the final statistical count is reduced.

When the initial number of fractures is 16, the internal defects are quite densely distributed. The spacing between fractures is very small. At the initial stage of loading, the stress fields around each tip interfere with one another. Once microcracks initiate, they quickly coalesce with the surrounding fractures. They form a small number of dominant large cracks. The number of independently dispersed new microcracks is very limited. Therefore, the final crack count is the lowest.

The growth rates of the three curves are similar. This indicates that, under the current simulation conditions, the number of initial fractures has no significant influence on the initiation rate of microcracks. Its main effect is reflected in the spatial range of crack initiation and the coalescence pattern. Fewer initial fractures provide more space for crack initiation, resulting in a larger final crack count. More initial fractures compress the available space for initiation. Cracks are more prone to coalescence, leading to a smaller final crack count.

(4) Presence or absence of prefabricated fractures (with fractures vs. without fractures)

From the final crack counts in Group (d) of Figure 4, it can be observed that the fracture-free specimen has the highest number of cracks. The fractured specimen has the lowest number of cracks. In terms of the crack growth rate, differences are observed at different loading stages. At the early stage of loading, the fractured specimen shows a faster rate than the fracture-free specimen. At the later stage of loading, the fracture-free specimen surpasses the fractured specimen in growth rate.

For the fracture-free specimen, there is no macroscopic initial defect. At the early stage of loading, the entire cross-section is uniformly stressed. The stress level is not sufficient to trigger local microcracking. Therefore, the early crack initiation rate is relatively slow. The curve is relatively flat. As the load increases, a large number of uniformly distributed microcracks gradually develop within the internal particles and bonds. These cracks are scattered throughout the entire specimen. There is no pre-existing fracture to guide their propagation. Therefore, the crack count continues to accumulate.

At the later stage of loading, the material damage intensifies. A large number of microcracks initiate simultaneously. The growth rate increases rapidly. It surpasses that of the fractured specimen. The final cumulative crack count reaches the highest level.

For the fractured specimen, the opposite trend is observed. The presence of initial fractures causes strong stress concentration at the tips even at the early stage of loading. Therefore, microcracks quickly initiate from the fracture ends. The rate is significantly faster than that of the fracture-free specimen. During the middle stage, these early cracks rapidly coalesce with the initial fractures. They form a few dominant macroscopic cracks. The subsequent damage evolution is mainly concentrated on further opening, sliding, and extension of these existing cracks. The number of newly initiated independent microcracks decreases significantly.

Therefore, the crack count of the fractured specimen rises rapidly at the early stage. It then tends to reach saturation. The growth rate slows down noticeably at the later stage. The final total is eventually surpassed by that of the fracture-free specimen.

This comparison shows that the fractured specimen generates a large number of microcracks rapidly at the early stage. However, due to the initial defects, the damage path is highly concentrated. The potential for generating new cracks is quickly exhausted. In contrast, the fracture-free specimen lacks pre-existing weak planes to guide the damage. Therefore, the damage accumulates slowly in a uniformly distributed manner. It eventually produces the highest number of microcracks. This further indicates that the superior load-bearing capacity of the fracture-free specimen originates from the well-distributed damage throughout the entire specimen volume, rather than localised concentration in a specific region.

(5) Comprehensive analysis and discussion

By synthesising the four groups of crack number evolution curves, it can be concluded that the final crack count does not directly represent the severity of specimen degradation. In the inclination angle group, the 60° specimen has the most cracks but shows the highest load-bearing capacity. In the length group, the short-fracture specimen has the most cracks but shows the least degradation. In the number group, the specimen with 4 initial fractures has the most cracks but shows the highest load-bearing capacity. In the presence-or-absence group, the fracture-free specimen has the most cracks but shows the best mechanical performance.

The key factor governing the macroscopic mechanical performance is whether the microcracks are uniformly dispersed or concentrated into a through-going fracture. The crack evolution laws from the PFC simulations and the stress–strain curve laws mutually support each other. Together, they reveal the internal mechanism by which initial fracture defects regulate the initiation location, propagation path, and coalescence mode of damage, thereby influencing the macroscopic failure mode of concrete.

#### 4.1.3. Load–Displacement Curves

During the uniaxial compression loading process, the load sustained by the specimen continuously varies with the applied displacement. The peak load, the displacement corresponding to the peak, and the rate of curve change collectively reflect the load-bearing capacity, deformability, and failure intensity of concrete. Based on multiple sets of load–displacement curves obtained from PFC numerical simulations, this study examines the influence of initial fracture characteristics on the load response of concrete from four aspects. These are fracture inclination angle (30°, 45°, and 60°), fracture length (short, long, and very long), number of fractures (4, 8, and 16), and the presence or absence of prefabricated fractures. The displacements include three components: particle slip, shear displacement, and the total displacement resulting from material compression.

The load–displacement curves under different test conditions show obvious differences in peak load, ultimate displacement, and rate of change. The overall trend is as follows. A larger fracture inclination angle, a shorter fracture length, and a smaller number of fractures all result in higher load values, larger ultimate displacement, and faster rates of change. The fracture-free specimen outperforms the fractured specimen in all aspects. Detailed analyses for each case are presented below.

(1) Fracture inclination angle (30°, 45°, 60°)

**Figure 5:**
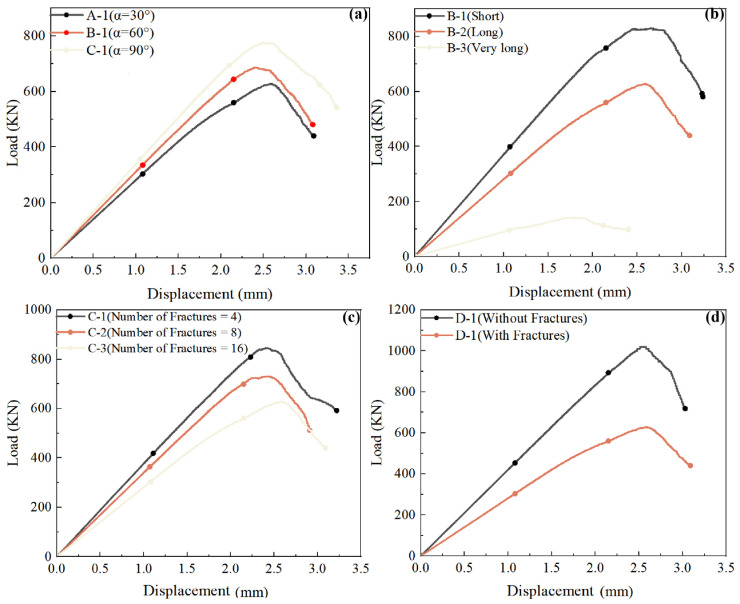
Load–displacement curves for specimens under different conditions: (**a**) fracture inclination angle; (**b**) fracture length; (**c**) number of fractures; (**d**) presence or absence of prefabricated fractures.

From Group (a) in Figure 5, it can be observed that the specimen with a 60° inclination angle has the highest peak load. It also has the largest ultimate displacement. Its rate of curve change is the fastest. In contrast, the 30° specimen shows the lowest peak load. It also has the smallest ultimate displacement. Its rate of curve change is the slowest. The 45° specimen falls between the two.

Regarding the peak load, the following trend is observed. For a large inclination angle, the fracture plane tends to close under compression. The tensile stress concentration at the tip is relatively weak. The overall stress distribution in the specimen is more uniform. Therefore, it can sustain a higher ultimate load. For a small inclination angle, the fracture plane is nearly horizontal. The shear effect on the fracture surface is significant. Cracks initiate at the tips and propagate rapidly even at a relatively low load. This leads to premature structural failure.

Regarding the ultimate displacement, the 60° specimen can still maintain a certain load-bearing capacity after reaching the peak load. The deformation process lasts longer. This reflects better ductility characteristics. In contrast, the 30° specimen shows a sharp drop in load once the peak is reached. Its deformation capacity is limited.

Regarding the rate of change, the load–displacement curve of the 60° specimen has the steepest slope in the ascending branch. This indicates that its initial stiffness is the highest. The structural response under external loading is rapid. The 30° specimen shows a gentler slope. Its stiffness degradation is significant.

(2) Fracture length (short, long, and very long)

From Group (b) in Figure 5, it can be observed that the short-fracture specimen has the highest peak load. It also has the largest ultimate displacement. Its rate of curve change is the fastest. In contrast, the very-long-fracture specimen shows the lowest peak load. It also has the smallest ultimate displacement. Its rate of curve change is the slowest. The long-fracture specimen falls between the two.

Regarding the peak load, the following trend is observed. Short fractures cause limited cutting of the specimen cross-section. The effective load-bearing area remains relatively large. The stress distribution is relatively uniform. Therefore, the ultimate load is the highest. As the fracture length increases, the effective bearing cross-section continuously decreases. The high-stress zone at the fracture tips expands. Microcracks are more likely to initiate from the tips and coalesce. This leads to specimen failure at a relatively low load.

Regarding the ultimate displacement, the short-fracture specimen undergoes a complete sequence of elastic, yield, and failure stages during loading. The deformation is fully developed. In contrast, the very-long-fracture specimen is almost divided into two independent parts by the fracture. There is no complete load-transfer skeleton during loading. Once the peak load is reached, the specimen immediately loses stability. Its deformation capacity is extremely limited.

Regarding the rate of change, the short-fracture specimen has the steepest ascending branch. This indicates that its initial stiffness is the highest. The very-long-fracture specimen suffers from severe initial defects. Its overall stiffness degradation is significant. The curve shows the most gentle change.

(3) Number of fractures (4, 8, and 16)

From Group (c) in Figure 5, it can be observed that the specimen with 4 initial fractures has the highest peak load. It also has the largest ultimate displacement. Its rate of curve change is the fastest. In contrast, the specimen with 16 fractures shows the lowest peak load. It also has the smallest ultimate displacement. Its rate of curve change is the slowest. The specimen with 8 fractures falls between the two.

Regarding the peak load, the following trend is observed. The specimen with 4 fractures has fewer internal defect sites. The stress fields around each fracture tip are independent of one another. The matrix material can still form a relatively continuous overall load-bearing framework. Therefore, the ultimate load is the highest. The specimen with 16 fractures has densely distributed internal defects. The stress fields between fractures overlap with each other. The weak zones are connected into a continuous area. The external force cannot be effectively transmitted through an intact path. The ultimate load is significantly reduced.

Regarding the ultimate displacement, the 4-fracture specimen undergoes a relatively long deformation process from loading to failure. The descending branch of the curve is relatively gentle. This reflects a certain degree of ductility. The 16-fracture specimen shows a sharp drop in load after reaching the peak. This indicates that its failure mode is more brittle.

Regarding the rate of change, the 4-fracture specimen has the highest initial overall stiffness. The ascending branch of the curve is the steepest. The 16-fracture specimen suffers from the most severe overall stiffness degradation due to the dense distribution of internal damage zones. The curve shows the most gentle change.

(4) Presence or absence of prefabricated fractures (with fractures vs. without fractures)

From Group (d) in Figure 5, it can be observed that the fracture-free specimen has the highest peak load. It also has the largest ultimate displacement. Its rate of curve change is the fastest. In contrast, the fractured specimen shows the lowest peak load. It also has the smallest ultimate displacement. Its rate of curve change is the slowest.

Regarding the peak load, the fracture-free specimen has a continuous and intact internal structure. The external force is uniformly transmitted across the entire cross-section. There is no initial weak plane. Therefore, the ultimate load is significantly higher than that of the fractured specimen. The fractured specimen exhibits stress concentration at the fracture edges. Local damage occurs even at the early stage of loading. The load-bearing capacity is clearly limited.

Regarding the ultimate displacement, the damage in the fracture-free specimen accumulates gradually in a uniformly distributed manner. It undergoes a complete sequence of elastic, yield, and strain-hardening stages before failure. The deformation is fully developed. The fractured specimen has its failure path guided by initial defects. The damage is highly concentrated. Its deformation capacity is limited. Once the peak load is reached, it quickly loses stability.

Regarding the rate of change, the fracture-free specimen has the steepest ascending branch in the load–displacement curve. Its initial stiffness is the highest. The fractured specimen suffers from initial structural discontinuity. Its overall stiffness is significantly reduced. The curve shows a relatively gentle change.

(5) Comprehensive analysis and discussion

By synthesising the four groups of load–displacement curves, it can be concluded that the three indicators—peak load, ultimate displacement, and rate of change—show a completely consistent order across all test conditions. All point to the same pattern. A larger fracture inclination angle, a shorter fracture length, and a smaller number of fractures all lead to better load-bearing capacity, deformability, and overall stiffness. The fracture-free specimen outperforms the fractured specimen in all indicators.

The results of the load–displacement curves under each test condition mutually confirm the findings from the stress–strain curve analysis. Together, they demonstrate that initial fracture defects weaken the effective load-bearing area, intensify stress concentration, and disrupt the load transfer path. At the macroscopic level, these effects manifest as reduced load, decreased displacement, and degraded stiffness.

### 4.2. Crack Count and Morphological Changes

#### 4.2.1. Fracture Inclination Angle (30°, 45°, 60°)

(1) Low inclination prefabricated fracture (30°)

**Figure 6:**
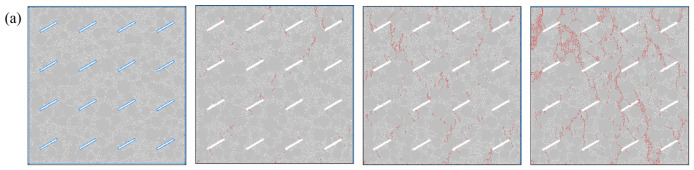

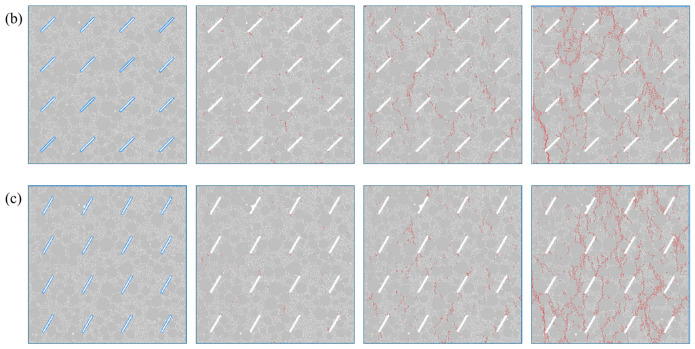
Crack propagation process in specimens with different fracture inclination angles: (**a**) 30°; (**b**) 45°; (**c**) 60°.

From Group (a) in Figure 6, the following observations are made. At the initial stage, small stress concentration zones exist at the edges of the prefabricated fractures. The mortar matrix remains intact. No new cracks are generated. As the load increases, new cracks first initiate from both ends of the prefabricated fractures. The propagation direction is roughly the same as the fracture orientation. At the early stage, the new cracks are separated from each other. They only develop locally around each prefabricated fracture. The number of cracks is small. Their length is limited.

As the load continues to increase, the cracks derived from the prefabricated fractures gradually extend into the mortar matrix. The cracks between adjacent fractures begin to overlap and connect with each other. Some discontinuously distributed crack branches appear in the middle of the specimen. The overall distribution is still relatively sparse.

After reaching the peak load, the specimen enters the residual damage stage. A large number of branched cracks interconnect and intertwine with each other. They form multiple oblique main fracture zones that run through the specimen. The fracture network covers most of the specimen area. The internal damage of the specimen is fully developed.

(2) Medium inclination prefabricated fracture (45°)

From Group (b) in Figure 6, the following observations are made. At the initial compression stage, the overall structure of the specimen remains intact. Stress concentration may exist at the tips of each prefabricated fracture. However, no obvious new damage is observed. As the load increases slightly, new cracks simultaneously initiate at the ends of all prefabricated fractures. The initiation direction of these cracks is consistent with the inclination angle of the fractures. At the early stage, the cracks are dispersed around each fracture. They are not connected to one another.

As loading continues, microcracks rapidly extend into the interior of the matrix. A large number of cracks derived from different fractures overlap and connect with each other. The crack density increases significantly. Patches of cracks appear in both the upper and lower regions of the specimen. Small through-going cracks have already formed locally. When the load approaches the peak value, a large number of secondary cracks initiate and propagate along the main fractures. Oblique main cracks completely penetrate the specimen. The interwoven fracture network becomes denser. The overall degree of crack connectivity is higher than that in the low-inclination-angle case.

(3) High inclination prefabricated fracture (60°)

From Group (c) in Figure 6, the following observations are made. At the initial stage of loading, the mortar matrix shows no damage. Stress concentration exists at the tips of the prefabricated fractures, but no new cracks are generated. When the load reaches a certain level, microcracks rapidly initiate in batches at the ends of each prefabricated fracture. The new cracks extend from the tips into the matrix at a faster speed. Within a short period, local crack overlapping occurs between adjacent fractures in multiple locations.

As loading continues, a large number of branched fractures develop simultaneously throughout the entire specimen. The crack count increases significantly. Continuous crack belts form in both the central and marginal regions. The distribution is uniform and dense. Upon entering the failure stage, the criss-crossing fractures completely coalesce. The main crack penetrates the entire specimen along the inclination direction of the prefabricated fractures. Secondary cracks are widely distributed throughout the mortar matrix. The internal damage range is the largest. The degree of fragmentation is the most obvious. The fracture network is more fully developed than in the other two inclination angle cases.

#### 4.2.2. Fracture Length (Short, Long, and Very Long)

(1) Short prefabricated fracture specimen

**Figure 7:**
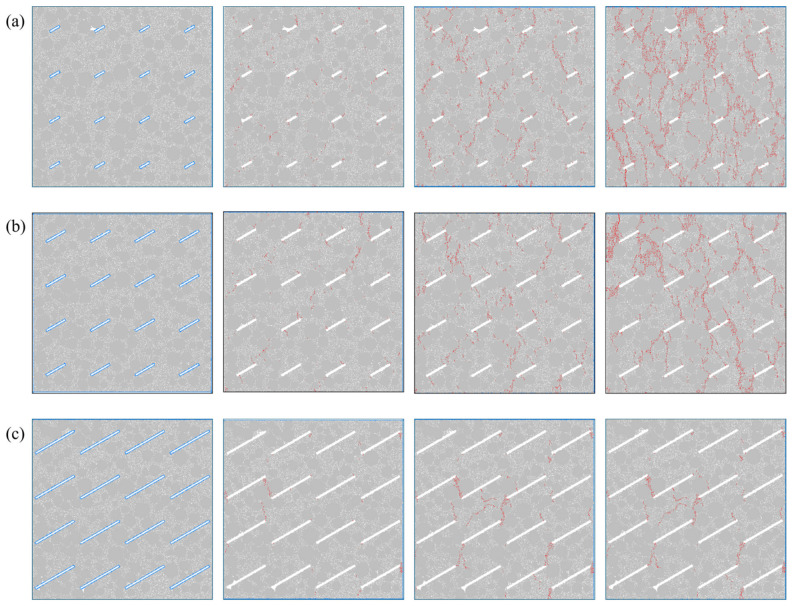
Crack propagation process for specimens under different fracture lengths: (**a**) short; (**b**) long; (**c**) very long.

From Group (a) in Figure 7, the following observations are made. At the initial stage of loading, the specimen matrix remains intact. Stress concentration only exists at the tips of each short prefabricated fracture. No new microcracks are generated. As the load increases slightly, a large number of microcracks simultaneously initiate from both ends of each short fracture. The newly generated fine cracks rapidly extend into the surrounding mortar matrix.

During continuous compression, a single short fracture induces multiple branched cracks. The cracks derived from adjacent short fractures easily overlap and connect with each other. Damage develops simultaneously throughout the entire specimen. The crack density continues to increase rapidly. At the final stage of loading, a large number of interlaced and coalesced branched cracks cover the entire specimen area. They form an extremely dense network-like damage structure. This case has the largest total number of new cracks and the widest fragmentation range among the three length groups.

(2) Long prefabricated fracture specimen

From Group (b) in Figure 7, the following observations are made. At the initial stage of loading, stress concentration only exists at the tips of the elongated prefabricated fractures. As the load increases, microcracks initiate from both ends of the long fractures. They extend over longer distances. However, the number of branches induced by a single prefabricated fracture is less than that in the short fracture case. During the middle stage of loading, discontinuous connected crack paths gradually form between different long fractures. Obvious damage cracks can be observed throughout the entire specimen. At the final stage, although an oblique through-going main fracture zone is formed, the density of crack interweaving and the total crack number are both lower than those in the short-fracture specimen. The intact matrix regions are more extensive than those in Group (a).

(3) Very long inclination prefabricated fracture (60°)

From Group (c) in Figure 7, the following observations are made. The stress concentration at the tips of the very long prefabricated fractures is significant. However, after crack initiation, the propagation direction of new cracks is relatively uniform. Most cracks develop along the direction parallel to the prefabricated fractures. The fractures themselves are long in length. The spacing between adjacent fractures is small. It is difficult for cracks to overlap and connect across a large range between fractures. Even at the final stage of loading, only a small number of connected cracks appear in local regions of the specimen. The total number of new cracks is far less than that in the previous two groups. Most of the mortar matrix shows no obvious damage. The overall degree of fragmentation is the lowest.

#### 4.2.3. Number of Fractures (4, 8, and 16)

(1) Specimen with 4 prefabricated fractures

**Figure 8:**
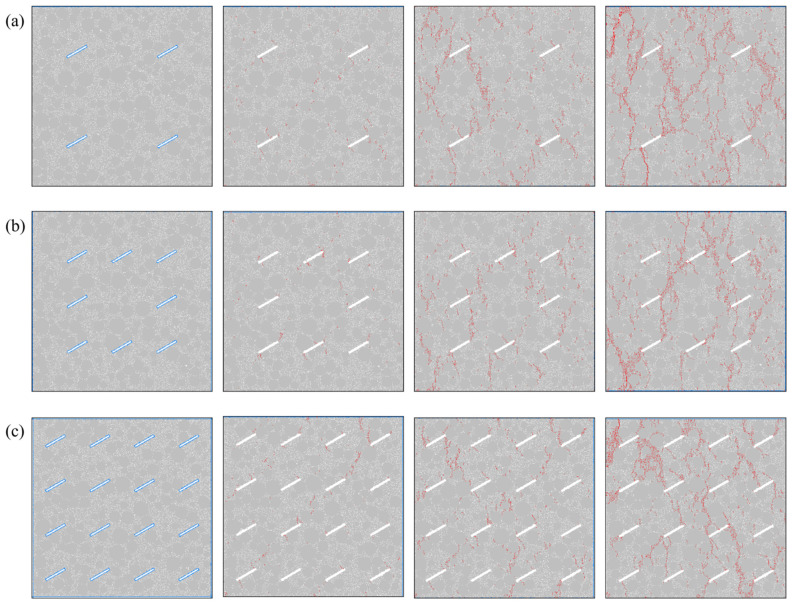
Crack propagation process for specimens under different numbers of fractures: (**a**) 4; (**b**) 8; (**c**) 16.

From Group (a) in Figure 8, the following observations are made. At the initial stage of loading, the matrix inside the specimen remains intact. Stress concentration only exists at the tips of the four prefabricated fractures. No new red cracks are observed. As the load increases gradually, microcracks initiate from both ends of each prefabricated fracture. They gradually extend to farther regions.

As the load continues to increase, the main cracks derived from the four prefabricated fractures continuously expand outward. They extend all the way to the upper, lower, left, and right edges of the specimen. At the same time, these cracks intertwine with each other in the central region of the specimen. They form a complete fracture network. Some cracks converge into thicker cracks. Others aggregate into a mesh-like distribution. Finally, the fracture network covers most of the specimen area. The damage range is the largest. The overall degree of fragmentation is the most significant.

(2) Specimen with 8 prefabricated fractures

From Group (b) in Figure 8, the following observations are made. At the initial compression stage, eight prefabricated fractures are uniformly distributed in the specimen. Stress concentration exists at the tips of each fracture. However, the matrix shows no obvious damage for the time being. As the load increases, microcracks initiate at the tips of multiple fractures. However, the propagation directions of these cracks are not uniform. Few cracks develop in the upper-left region. The cracks mainly extend toward the lower-left direction. Only a small number of cracks derived from adjacent prefabricated fractures connect with each other. The degree of connectivity is relatively low. In addition, some short and small cracks are scattered around each prefabricated fracture. However, the overall distribution is relatively dispersed.

During the middle and late stages of loading, some cracks are still present locally. However, both the extension range and the degree of connectivity are significantly weaker than those in the 4-fracture case. Finally, the coverage range and penetration degree of the fracture network are the smallest among the three groups.

(3) Specimen with 16 prefabricated fractures

From Group (c) in Figure 8, the following observations are made. At the initial stage of loading, although the number of prefabricated fractures is large, the appearance of new cracks is relatively slow. Only a small number of microcracks slowly initiate from the ends of each prefabricated fracture. As the load continues to increase, the specimen enters the middle stage. Some prefabricated fractures become connected through extended cracks. Local connectivity is formed.

As the load further increases, cracks gradually extend toward the upper-left and lower-right directions. Finally, a large oblique through-going crack penetrates the specimen. At the same time, cracks also branch and extend in a “Y”-shaped pattern. A fracture network forms in the upper-left region. Overall, the degree of fracture development in this case is higher than that in the 8-fracture case. However, it is lower than that in the 4-fracture case. The connectivity range and network density are intermediate between the two.

#### 4.2.4. Presence or Absence of Prefabricated Fractures (With Fractures vs. Without Fractures)

(1) Specimen with uniformly distributed prefabricated fractures

**Figure 9:**
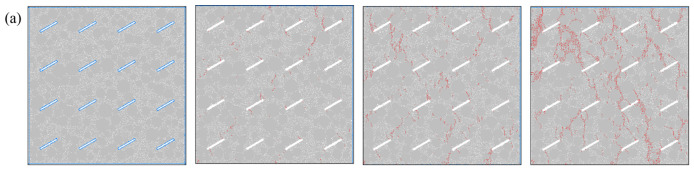

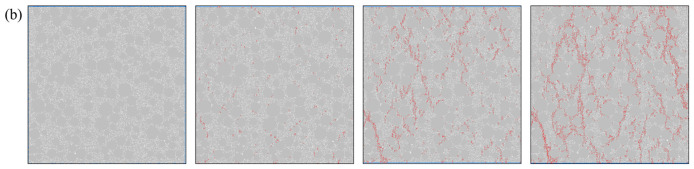
Crack propagation process for specimens with and without prefabricated fractures: (**a**) with fractures; (**b**) without fractures.

From Group (a) in Figure 9, the following observations are made. At the initial stage of loading, the specimen matrix remains intact. Stress is only concentrated at both ends of each prefabricated fracture. No new red cracks appear inside the matrix. As the load increases slightly, microcracks simultaneously initiate at the tips of all prefabricated fractures. The new damage slowly extends from the crack tips into the mortar matrix. The propagation direction deviates from the orientation of the prefabricated fractures. The cracks are scattered around each fracture.

During continuous loading, the cracks derived from adjacent prefabricated fractures gradually overlap and connect with each other. Multiple discontinuously connected damage zones form inside the specimen. The cracks are mainly concentrated in the regions where the prefabricated fractures are arranged. Other parts of the matrix show relatively minor damage. At the final stage of failure, the prefabricated fractures guide the formation of multiple oblique main cracks. The overall distribution of the crack network is controlled by the arrangement of the prefabricated fractures. The damaged zones are distributed in banded patterns along the fracture orientation. The overall crack arrangement is relatively regular.

(2) Intact specimen without prefabricated fractures

From Group (b) in Figure 9, the following observations are made. At the initial compression stage, there are no prefabricated defects inside the specimen. No obvious local stress concentration exists. Only when the load increases to a certain level do some fine microcracks randomly appear at the interfaces between mortar and aggregate. The locations of crack initiation have no fixed pattern. They are uniformly scattered throughout the entire specimen.

As the load continues to increase, the interfacial microcracks continuously extend and branch. The cracks at different locations freely overlap and intertwine with each other. They are not constrained by any preferential direction. They develop simultaneously in the upper, lower, left, and right regions of the specimen. At the final failure stage, a dense network-like fracture system forms throughout the specimen without any obvious orientation. The fractured cracks are randomly distributed in a criss-crossing pattern. There is no main fracture zone developing along a fixed direction. The overall damage is distributed across the entire specimen. The crack distribution shows no obvious zoning characteristics.

## 5. Discussion

### 5.1. Effect of Prefabricated Fracture Inclination Angle on Crack Propagation

Under uniaxial compression, the inclination angle between the prefabricated fracture and the horizontal direction is a key factor affecting crack propagation in concrete. This study uses PFC discrete element simulations. It compares and analyses the mechanical responses and crack evolution patterns under three inclination angles: 30°, 45°, and 60°. Overall, a larger inclination angle leads to better load-bearing capacity and deformability. However, it also produces a greater total number of new cracks. A smaller inclination angle causes earlier crack initiation and faster coalescence. The failure is more sudden. This was also mentioned in the paper by Taito Miura et al. [39]. At small inclination angles, that is, when the angle is between 0 and 60 degrees, the crack intersects with the loading axis, and shear deformation causes micro-cracks to form, resulting in a decrease in the bearing capacity. At a large inclination angle, that is, when the angle is 90°, the cracks are parallel to the loading axis, and there are fewer micro-cracks, resulting in a relatively higher load-bearing capacity.

From the perspective of macroscopic mechanical properties, the peak stress of the 60° specimen is approximately 15% to 20% higher than that of the 30° specimen. The 60° specimen also has higher initial stiffness and better post-peak deformability. The reasons are as follows. For a small inclination angle, the fracture plane is nearly horizontal. The shear stress component on the fracture surface is relatively large. Significant tensile stress concentration occurs at the crack tips. Microcracks initiate and propagate rapidly even at a low stress level. This leads to a rapid reduction in the effective load-bearing area. For a larger inclination angle, the fracture plane tends to close under compression. The tensile effect at the tips is weakened. The external force is transmitted more uniformly. Therefore, the specimen can sustain a higher load.

Further observation of the crack number evolution curves reveals a noteworthy phenomenon. The 30° specimen shows the fastest crack initiation rate at the early stage. However, it has the lowest final cumulative crack count. The 60° specimen shows the opposite trend. It has the slowest initiation rate at the early stage but the highest final crack count. This difference arises from the different damage evolution paths under different inclination angles. For the 30° case, microcracks initiate at the fracture tips even at a low stress level. They propagate and coalesce rapidly along the dominant direction. The specimen loses structural stability within a relatively short period. The loading duration is short. There is insufficient time for more new cracks to initiate. Therefore, the final total is the lowest. For the 60° case, the tensile stress concentration at the tips is weaker. Microcracks require a higher stress level to initiate. The early-stage rate is slow. However, the large-inclination specimen can sustain a higher load and undergo a longer deformation process. Damage spreads in multiple directions. The initiation rate increases significantly in the middle stage. The final cumulative crack count becomes the highest. The 45° specimen shows intermediate behaviour in all aspects.

From the perspective of final failure morphology, the fracture networks also show obvious differences under different inclination angles. For the 30° case, the number of cracks is relatively small. However, the cracks are strongly directional. The main fracture zone runs diagonally through the specimen along the prefabricated fracture orientation. The degree of connectivity is high. This indicates brittle shear failure characteristics. The 60° case is different. A large number of branched cracks develop simultaneously throughout the entire specimen. Continuous crack belts form in both the central and marginal regions. The distribution is uniform and dense. Although the fragmentation range is the widest, no highly concentrated through-going main fracture surface is formed. The failure mode is relatively moderate. The 45° case shows intermediate levels of fracture network density and connectivity.

Based on the above analysis, it can be concluded that when evaluating the stability of concrete containing fractures, the final crack count alone should not be used to judge the degree of degradation. Small-inclination fractures generate fewer cracks. However, they have fast coalescence, concentrated directionality, and strong suddenness of failure. They pose a greater threat to structural safety. Large-inclination fractures induce a large number of microcracks. However, these cracks are dispersed. The formation of macroscopic through-going fractures is delayed. The specimen actually shows higher load-bearing capacity. This understanding suggests that in practical engineering inspections, special attention should be paid to fractures with small angles to the loading direction. These are important hidden dangers that can trigger sudden failure.

### 5.2. Effect of Prefabricated Fracture Length on Crack Propagation

The length of a prefabricated fracture directly determines the degree of weakening of the effective load-bearing cross-section. It also determines the size of the stress concentration zone at the crack tip. It is an important geometric factor affecting crack propagation. This study uses PFC discrete element simulations. It compares and analyses the mechanical responses and crack evolution patterns under three length conditions: short, long, and very long. Overall, a shorter fracture leads to higher load-bearing capacity, more new cracks, and a denser crack network. A longer fracture causes a more significant reduction in load-bearing capacity. However, it produces fewer new cracks. The failure mode becomes more brittle. P. Stroeven [40] noted in his paper that the crack length is positively correlated with the looseness of the structure.

From the perspective of macroscopic mechanical properties, the peak stress of the short-fracture specimen is approximately 25% to 30% higher than that of the long-fracture specimen. The peak stress reduction for the very-long-fracture specimen even exceeds 40%. Short fractures have a limited cutting effect on the matrix. The stress concentration zone at the tip is small. A large amount of intact matrix around the tip can effectively constrain microcrack propagation. As the fracture length increases, the effective bearing area decreases. The high-stress zone at the tip expands. The external force can only be transmitted through the narrow regions at the fracture ends. Once microcracks initiate, they quickly coalesce. The specimen exhibits obvious brittle failure characteristics.

From the crack number evolution curves, the short-fracture specimen has the highest final crack count and the fastest initiation rate. The very-long-fracture specimen has the lowest final count and the slowest rate. This trend appears counterintuitive. A longer fracture causes more severe structural weakening. However, it induces fewer new cracks. The reasons are as follows. For short fractures, microcracks initiate at the tips. They are continuously hindered by the surrounding intact matrix during propagation. They cannot easily coalesce. The loading energy is continuously converted into dispersed new cracks. Therefore, the crack count accumulates to a relatively high level. For long fractures, the high-stress zone at the tip is larger. Once microcracks initiate, they rapidly extend along the tip direction and connect with the main fracture. The total number of new cracks is relatively small. For very long fractures, the fracture almost runs through the entire specimen. The internal structural continuity is severely damaged. During loading, damage is dominated by further opening and sliding of the existing fracture. It is difficult to initiate a large number of new independent microcracks. Therefore, the crack count increases slightly at the initial stage and then tends to stagnate.

From the perspective of final failure morphology, the three length conditions also show obvious differences. In the short-fracture specimen, a large number of branched cracks simultaneously initiate from both ends of each fracture. They extend into the surrounding matrix. Cracks derived from adjacent fractures easily overlap and connect. They finally form an extremely dense network-like damage structure. The fragmentation range is the largest. The long-fracture specimen forms an oblique through-going main fracture zone. However, the density of crack interweaving and the total crack number are both lower than those in the short-fracture case. The very-long-fracture specimen shows the most limited damage. The propagation direction of new cracks is relatively uniform. Most cracks develop parallel to the fracture orientation. It is difficult for cracks to overlap across a large range between fractures. Most of the mortar matrix shows no obvious damage.

In summary, the effect of fracture length on crack propagation is reflected at two levels. At the macroscopic level, a longer fracture reduces the effective bearing area and intensifies stress concentration. The load-bearing capacity and deformability become worse. At the mesoscopic level, short fractures continuously activate a large number of new cracks and form a dense damage network. Long fractures concentrate damage at the tips. The number of new cracks is limited. However, the coalescence speed is fast and the failure is sudden. This understanding suggests that long fractures pose a more prominent threat to load-bearing capacity and should be given priority attention. Short fractures have relatively higher load-bearing capacity. However, they continuously induce new cracks during long-term service. This poses a potential threat to durability.

### 5.3. Influence of Different Numbers of Prefabricated Fractures on Crack Propagation

The number of prefabricated fractures determines the density of initial defects inside concrete. It also determines the degree of damage coupling effects. It is an important parameter affecting crack propagation. This study compares and analyses the mechanical responses and crack evolution patterns under three fracture number conditions: 4, 8, and 16. Overall, a smaller number of initial fractures leads to higher load-bearing capacity, more new cracks, and a wider fracture network coverage. A larger number of fractures causes a more significant reduction in load-bearing capacity. However, it produces fewer new cracks. Zhang et al. [41] discovered that fibers can inhibit the initiation of cracks, and the peak strength varies with the content of fibers, indicating that different initial crack densities have an impact on the bearing capacity.

From the perspective of macroscopic mechanical properties, the peak stress of the 4-fracture specimen is significantly higher than that of the 8-fracture and 16-fracture specimens. The peak stress of the 16-fracture specimen is only 60% to 70% of that of the 4-fracture specimen. Its post-peak curve drops sharply. The brittle characteristics are more pronounced. When only 4 fractures are present, the stress concentration zones around each tip are relatively independent. The matrix can still form a continuous load-transfer network. When the number increases to 8, the spacing between fractures decreases. The stress fields begin to overlap. Multiple crack sources are activated simultaneously and quickly coalesce. When the number reaches 16, the specimen interior is almost divided into many small independent blocks by the fractures. The continuous load-bearing system collapses. The external force can only be transmitted through the narrow bridging zones between fractures. The specimen enters the failure stage almost without a noticeable elastic phase.

From the crack number evolution curves, the final cumulative crack count is the highest for the 4-fracture specimen. The 8-fracture specimen ranks second. The 16-fracture specimen has the lowest count. However, the initiation rates of the three groups are roughly comparable throughout the entire loading process. For the 4-fracture case, the stress fields around each tip are independent of one another. Microcracks gradually develop within each affected zone. The total number increases linearly with strain. For the 8-fracture case, stress field superposition occurs in some regions. Once microcracks initiate, they coalesce with adjacent fractures more quickly. Some new cracks merge into larger ones. The final statistical count is reduced. For the 16-fracture case, the defects are quite densely distributed. The stress fields around each tip interfere with one another at the initial stage of loading. Once microcracks initiate, they quickly coalesce with the surrounding fractures. The number of independently dispersed new cracks is very limited. The final count is the lowest.

From the perspective of final failure morphology, cracks in the 4-fracture specimen initiate from both ends of each fracture. They extend to various edges of the specimen. They intertwine with each other in the central region. They form a complete fracture network. The damage range is the largest. For the 8-fracture specimen, crack extension is uneven. Few cracks develop in the upper-left region. The cracks mainly extend toward the lower-left direction. Only scattered short cracks appear around each fracture. The network coverage and degree of penetration are the smallest among the three groups. The 16-fracture specimen falls between the two. In the middle stage, some fractures become connected through extended cracks. In the later stage, cracks extend toward the upper-left and lower-right directions. They form a large oblique through-going crack. At the same time, cracks branch in a Y-shaped pattern. A fracture network forms in the upper-left region.

In summary, the effect of fracture number on crack propagation is mainly reflected in the spatial density of initial defects. Fewer fractures provide more matrix space around each defect. Microcracks can fully initiate and propagate independently. The fracture network coverage is wider. More fractures reduce the spacing between defects. The stress field interference becomes more severe. The space for damage evolution is compressed. It is worth noting that the 16-fracture specimen has the lowest final crack count. However, the initial defect density itself has already caused fundamental damage to structural continuity. Therefore, its macroscopic load-bearing capacity is the worst. This indicates that the direct weakening of structural continuity by initial fractures and the potential for new crack initiation during loading are two aspects that need to be considered separately.

### 5.4. Effect of the Presence or Absence of Prefabricated Fractures on Crack Propagation

The presence or absence of prefabricated fractures determines whether initial weak planes exist inside the concrete. This study compares the simulation results of fractured specimens with those of intact specimens without fractures. It analyses the influence of initial defects on crack propagation. Overall, the fracture-free specimen outperforms the fractured specimen in load-bearing capacity, deformability, and stiffness. It also produces more cracks with a more uniform distribution. The fractured specimen shows damage concentrated near the fractures due to tip stress concentration. Its failure is more sudden.

From the perspective of macroscopic mechanical properties, the peak stress of the fracture-free specimen is approximately 1.0 to 1.2 times that of the fractured specimen. It also has a longer elastic stage. The fracture-free specimen has no structural discontinuities. The external force is uniformly transmitted across the entire cross-section. There is no stress concentration. Therefore, it can remain intact at a higher stress level. The fractured specimen shows the opposite trend. Stress concentration occurs at the fracture edges even at a low stress level. This disrupts the uniform load-transfer system. The stress that the specimen can sustain at the same strain is significantly reduced.

From the crack number evolution curves, the fracture-free specimen has a higher final crack count than the fractured specimen. However, at the early stage of loading, the fractured specimen shows a faster crack initiation rate. It is then surpassed by the fracture-free specimen at the later stage. The fracture-free specimen has no local stress concentration at the early stage. Crack initiation is relatively slow. As the load increases, uniformly distributed microcracks gradually develop inside the specimen. There is no prefabricated fracture to guide their propagation. The cracks are scattered throughout the entire specimen. At the later stage, a large number of microcracks initiate. The final cumulative count reaches the highest level. The fractured specimen initiates microcracks from the fracture ends at the early stage. The rate is faster than that of the fracture-free specimen. However, these early cracks rapidly coalesce with the initial fractures. The number of subsequent new independent cracks decreases significantly. The final total is eventually surpassed.

From the perspective of final failure morphology, cracks in the fracture-free specimen show no obvious directional characteristics. They randomly initiate at the interfaces between mortar and aggregate. They extend, branch, and freely overlap with each other. They form a network-like fracture system without a preferential direction. There is no main fracture zone along a fixed direction. Damage is distributed throughout the entire specimen. In contrast, cracks in the fractured specimen propagate along the loading direction. They gradually overlap and connect between adjacent fractures. The damaged zones are distributed in banded patterns along the fracture orientation. They eventually form multiple oblique main cracks. The directionality and regularity are obvious.

In summary, the fractured specimen can quickly initiate microcracks at the early stage of loading. However, the damage path is highly concentrated. The potential for new cracks is quickly exhausted. The fracture-free specimen lacks pre-existing weak planes to guide the damage. The damage accumulates slowly in a uniformly distributed manner. It eventually produces more microcracks and shows higher load-bearing capacity. This indicates that the presence of initial defects not only directly reduces load-bearing capacity. More importantly, it changes the damage mode from uniformly dispersed to locally concentrated. This makes the failure more sudden.

### 5.5. Future Research Prospects

This paper completes mesoscopic cracking simulations of concrete containing array prefabricated fractures using PFC2D discrete element method. Nevertheless, batch numerical simulations of multiple working conditions suffer from limitations including long computational cycles, trial-and-error dependence for mesoscopic parameter calibration, and low efficiency in manual interpretation of massive damage data. Future research can deeply couple artificial intelligence technology with the PFC platform to construct an integrated research framework of “numerical simulation—data mining—intelligent prediction” [42,43,44]. First, a deep learning surrogate model can be built based on the dataset of stress–strain responses, crack evolution and failure patterns generated from multiple PFC simulation cases in this study. By adopting UNet and deep operator networks, rapid mapping between geometric parameters of prefabricated fractures and concrete peak compressive strength, spatial distribution of microcracks and failure modes can be realized [45,46,47], which reduces the time cost of a single simulation from several hours to seconds and enables high-throughput orthogonal analysis of various fracture parameters. Second, genetic algorithms and Bayesian neural networks can be introduced to intelligently invert the mesoscopic mechanical parameters of PFC, replacing the traditional trial-and-error calibration process and eliminating the coupling interference between parallel bond parameters of mortar and aggregates, so as to improve the matching accuracy between the mesoscopic model and macroscopic mechanical responses from laboratory tests. Meanwhile, computer vision algorithms can be combined to automatically identify crack cloud images exported by PFC and quantify the aggregation and coalescence characteristics of microcracks [48,49,50,51,52]. Interpretable machine learning models can be established to distinguish two damage modes (uniform dispersion and concentrated coalescence), further refining the quantitative criterion proposed in this study that “the spatial distribution of microcracks dominates mechanical degradation”. In the long run, physics-informed neural networks can be integrated to extend uniaxial compression simulations to multi-field coupling conditions such as freeze–thaw and dry–wet cycles. Combined with fracture data measured by industrial CT in practical engineering, an intelligent prediction framework can be trained to realize real-time prediction of bearing capacity and sudden brittle failure risk of defective concrete structures, providing an efficient intelligent analysis tool for structural disease diagnosis in engineering practice.

## 6. Conclusions

This study uses the PFC discrete element simulation method. It systematically investigates the effects of prefabricated fracture inclination angle, length, number, and presence/absence on crack propagation in concrete under uniaxial compression. The following main conclusions are drawn.

(1) A larger fracture inclination angle leads to better load-bearing capacity and deformability. However, it also produces a greater total number of new cracks. A smaller inclination angle causes earlier crack initiation and faster coalescence. The failure is more sudden. For small-inclination cases, damage is dominated by rapid concentrated coalescence. For large-inclination cases, damage tends to spread in a dispersed manner.

(2) A shorter fracture leads to higher load-bearing capacity, more new cracks, and a denser crack network. A longer fracture causes a more significant reduction in load-bearing capacity. However, it produces fewer new cracks. Short fractures continuously activate a large number of new cracks and form a dense damage network. Long fractures concentrate damage at the tips. The crack coalescence speed is fast and the failure is sudden.

(3) Fewer initial fractures lead to higher load-bearing capacity, more new cracks, and wider fracture network coverage. More initial fractures cause a more obvious reduction in load-bearing capacity. However, they produce fewer new cracks. The number of fractures mainly affects the spatial range of crack initiation and the coalescence pattern, rather than the initiation rate.

(4) The fracture-free specimen outperforms the fractured specimen in both load-bearing capacity and deformability. It also produces more cracks with a more uniform distribution. In the fractured specimen, damage concentrates near the fractures due to tip stress concentration. The failure is more sudden. In the fracture-free specimen, damage accumulates slowly in a uniformly dispersed manner.

(5) A comprehensive comparison of the four groups reveals that the final crack count does not directly represent the degree of specimen degradation. The key factor governing macroscopic mechanical performance is the spatial distribution of microcracks. When microcracks are evenly dispersed, the specimen can still maintain relatively high load-bearing capacity. When they coalesce into through-going cracks, the capacity drops sharply. Prefabricated fracture defects fundamentally change the failure mode of concrete. They do so by regulating the initiation location, propagation path, and coalescence mode of damage. In practical engineering inspections, special attention should be paid to fractures with small angles to the loading direction and long fractures. These are the main hidden dangers that can trigger sudden failure.

## Figures and Tables

**Table 1:** Mesoscopic parameters of the PFC model.

Parameters of Mortar		Parameters of Aggregates	
Emod (Pa)	$130\times 10^{8}$	Emod (Pa)	$555\times 10^{8}$
Pb_emod (Pa)	$130\times 10^{8}$	Pb_emod (Pa)	$555\times 10^{8}$
Pb_ten (Pa)	$5\times 10^{6}$	Pb_ten (Pa)	$20\times 10^{6}$
Pb_coh (Pa)	$6\times 10^{6}$	Pb_coh (Pa)	$25\times 10^{6}$
Pb_PFA (°)	45	Pb_PFA (°)	40
Kratio	2	Kratio	1.5
damp	0.7	damp	0.7

## Data Availability

The original contributions presented in this study are included in the article. Further inquiries can be directed to the corresponding authors.
